# Supplementary material for: Tight Junction ZO Proteins Maintain Tissue Fluidity, Ensuring Efficient Collective Cell Migration
Source: Adv Sci (Weinh). 2021 Aug 12;8(19):2100478. doi: 10.1002/advs.202100478 (PMC8498871; doi:10.1002/advs.202100478)
Supplement: Supplementary file 1 — Supporting Information [file ADVS-8-2100478-s001.pdf]

WILEY-VCH

© 2021 Wiley-VCH GmbH

ADVANCED SCIENCE
Open Access

Supporting Information

for *Adv. Sci.,* DOI: 10.1002/advs.202100478

Tight junction ZO proteins maintain tissue fluidity, ensuring efficient collective cell migration

Mark Skamrahl, Hongtao Pang, Maximilian Ferle, Jannis Gottwald, Angela Rübeling, Riccardo Maraspini, Alf Honigmann, Tabea A. Oswald, and Andreas Janshoff**

© 2021 Wiley-VCH GmbH

Supporting Information

Tight junction ZO proteins maintain tissue fluidity, ensuring efficient collective cell migration

Mark Skamrahl, Hongtao Pang, Maximilian Ferle, Jannis Gottwald, Angela Rübeling, Riccardo Maraspini, Alf Honigmann, Tabea A. Oswald, and Andreas Janshoff**

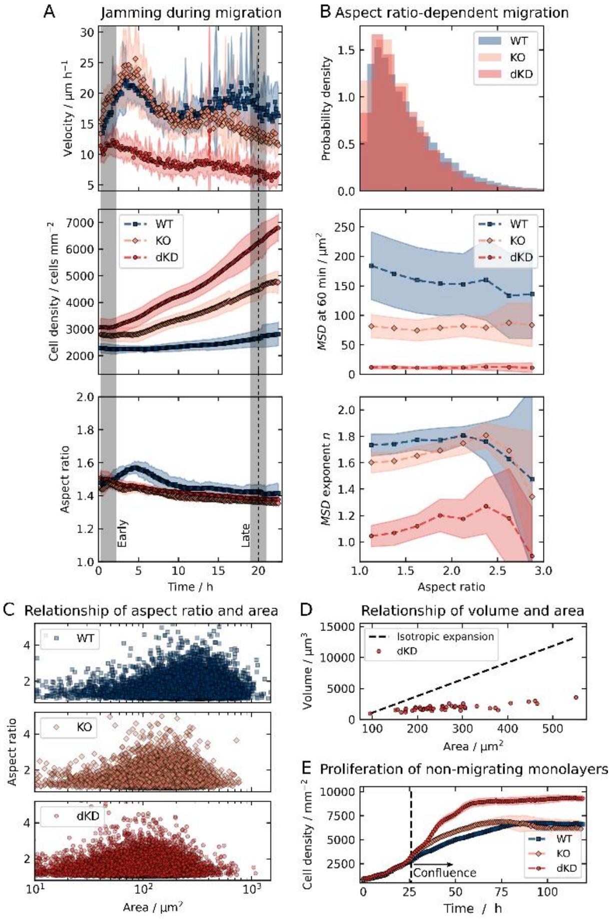

Figure S1. Temporal evolution of cell density, velocity, and aspect ratio as well as aspect ratio-dependent motility of all three untreated MDCK II cell lines. A) Cell crowding and jamming during migration as quantified by the velocity, cell density, and aspect ratio over time. The gray shades at 0.5-2.5 h ('early') and 19-21 h ('late') correspond to the time windows of the cell velocity and *MSD* analyses. B) Aspect ratio distribution and aspect ratio-dependent MSD parameters. C) The aspect ratio showed a high variance but no co-variation with the area. D) Relationship between the increase in cell volume with respect to the cell area in dKD cells determined from 3D-confocal actin stacks. For comparison, theoretical isotropic expansion is shown as a dashed line. E) Additional proliferation experiment immediately after seeding of cells without insert. The dashed line indicates reaching of confluence,

corresponding to the beginning of our typical migration experiments (0 h in all other figures). Means and standard deviations are shown. The aspect ratio in A is the median for each experiment and then averaged over all experiments.

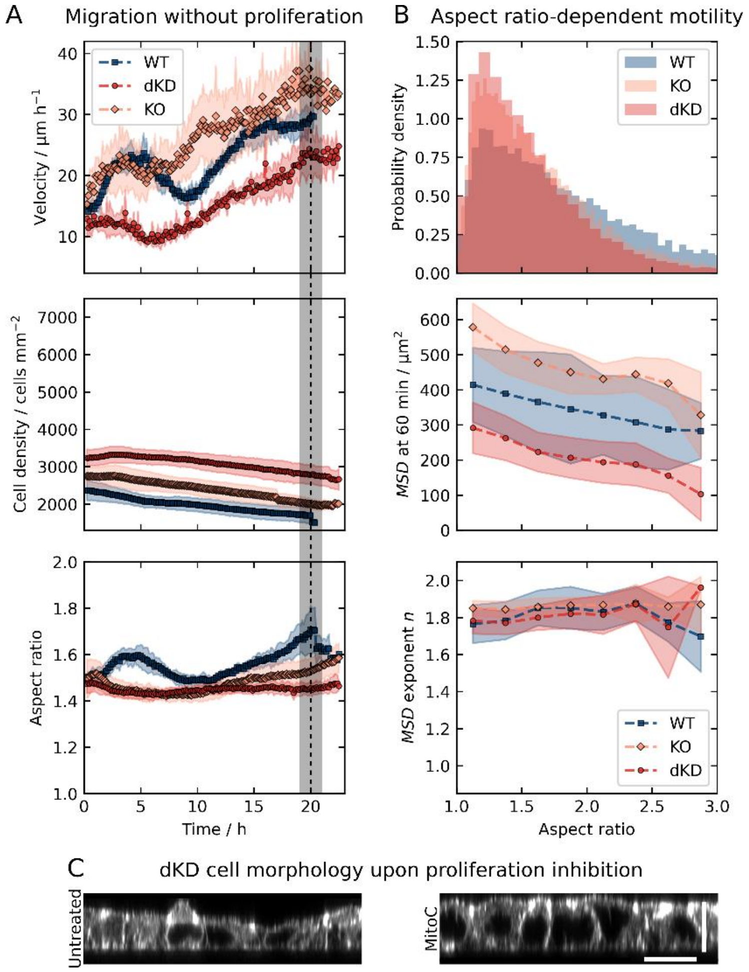

Figure S2. Temporal evolution of cell density, velocity, and aspect ratio as well as aspect ratio-dependent motility of all three MDCK II cell lines upon proliferation inhibition by Mitomycin C. A) Cell crowding and jamming were prevented by proliferation inhibiton during migration as quantified by the velocity, cell density, and aspect ratio over time. The gray shade at 19-21 h corresponds to the time window of the MSD analyses. B) Aspect ratio distribution and aspect ratio-dependent MSD parameters. Means and standard deviations are shown. The aspect ratio in A is the median for each experiment and then averaged over all experiments. C) Side-view of untreated and Mitomycin C treated dKD cells from 3D confocal actin stacks. Scale bars: 10 µm (z), 20 µm (x).

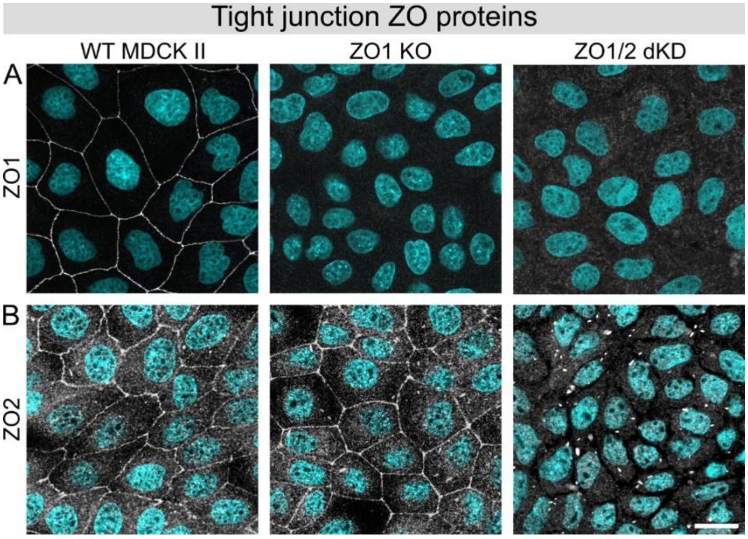

Figure S3. Immunofluorescence measurements confirming successful ZO protein knockout/-down. A) ZO1 antibody-based staining of all three MDCK II cell lines. B) ZO2 antibody-based staining of all three MDCK II lines. Nuclei are shown in cyan. Scale bar: 20 µm.

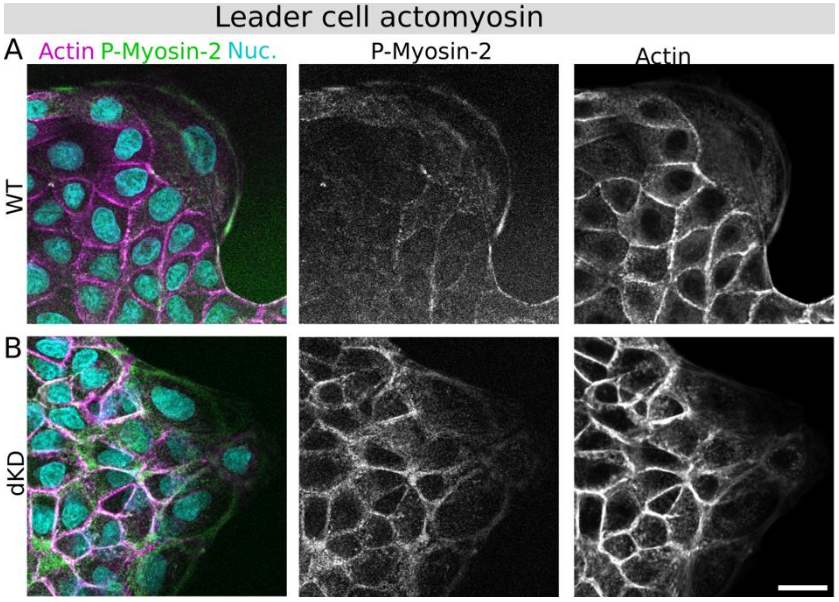

Figure S4. Actomyosin architecture remodeling for leader cells at the migration front of migrating WT and dKD cell layers. Phosphorylated Myosin-2 (P-Myosin-2; green), actin (magenta) and nuclei co-staining of MDCK II WT (A) and dKD (B) cell lines with corresponding gray-scale images of P-Myosin-2 and actin. Scale bar: 20 µm.

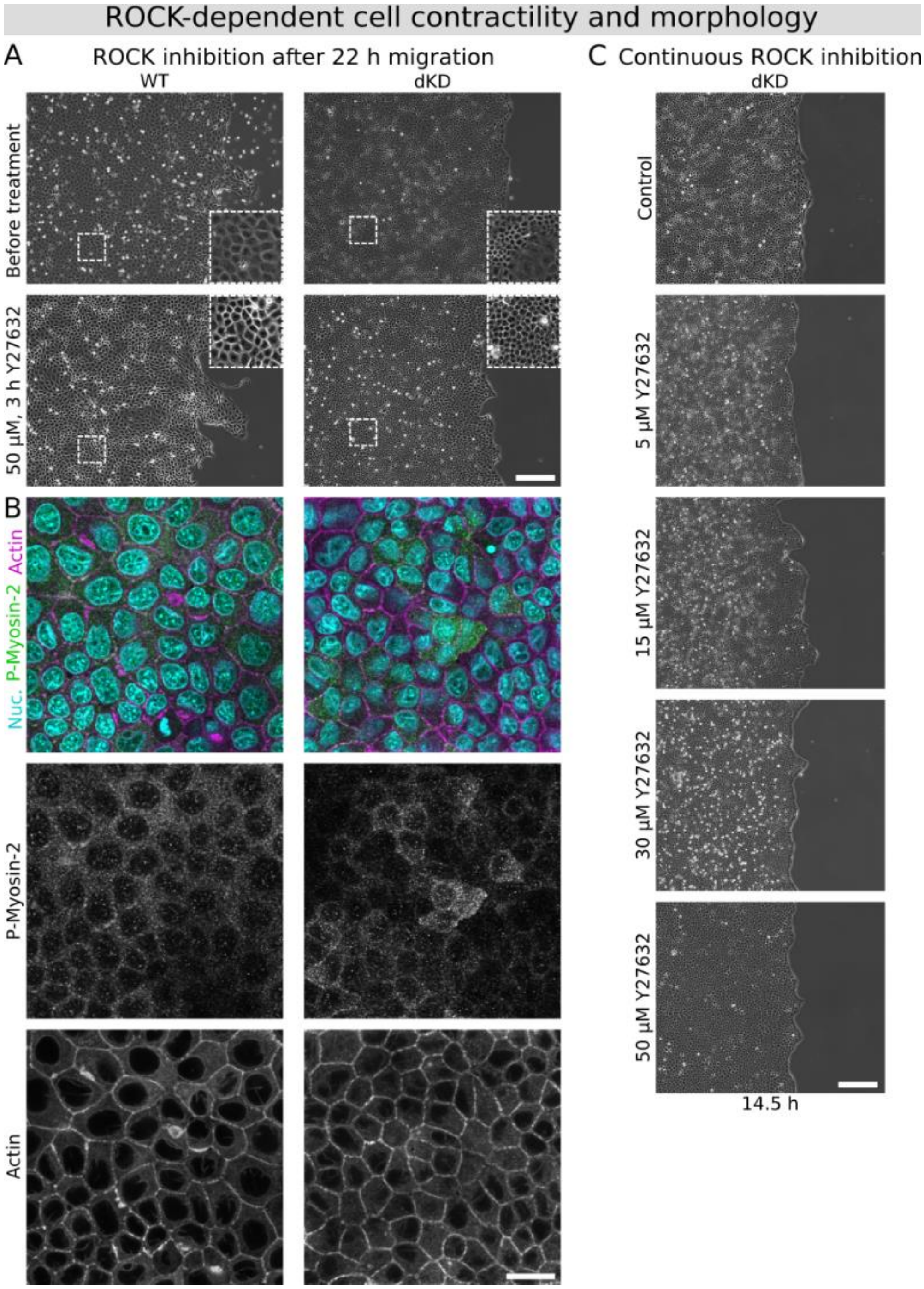

Figure S5. MDCKII WT and dKD cell lines show altered migration and actomyosin architecture remodeling upon inhibition of ROCK with Y27632. A) Inhibition of ROCK by Y27632 after 22 h of migration in WT and dKD cells. Top shows cell monolayers before treatment and bottom the same layers after 3 h of migration in the presence of 50 µM Y27632. Scale bar: 200 µm B) Confocal actomyosin images of the same MDCKII WT and dKD layers from A after Y27632 treatment after 22 h of migration with corresponding gray-scale images of P-Myosin-2 and actin. Scale bar: 20 µm. C) Migration of dKD cells for 14.5 h in the presence of increasing concentrations of Y27632 (starting continuously from 30 min before insert removal). Scale bar: 200 µm.

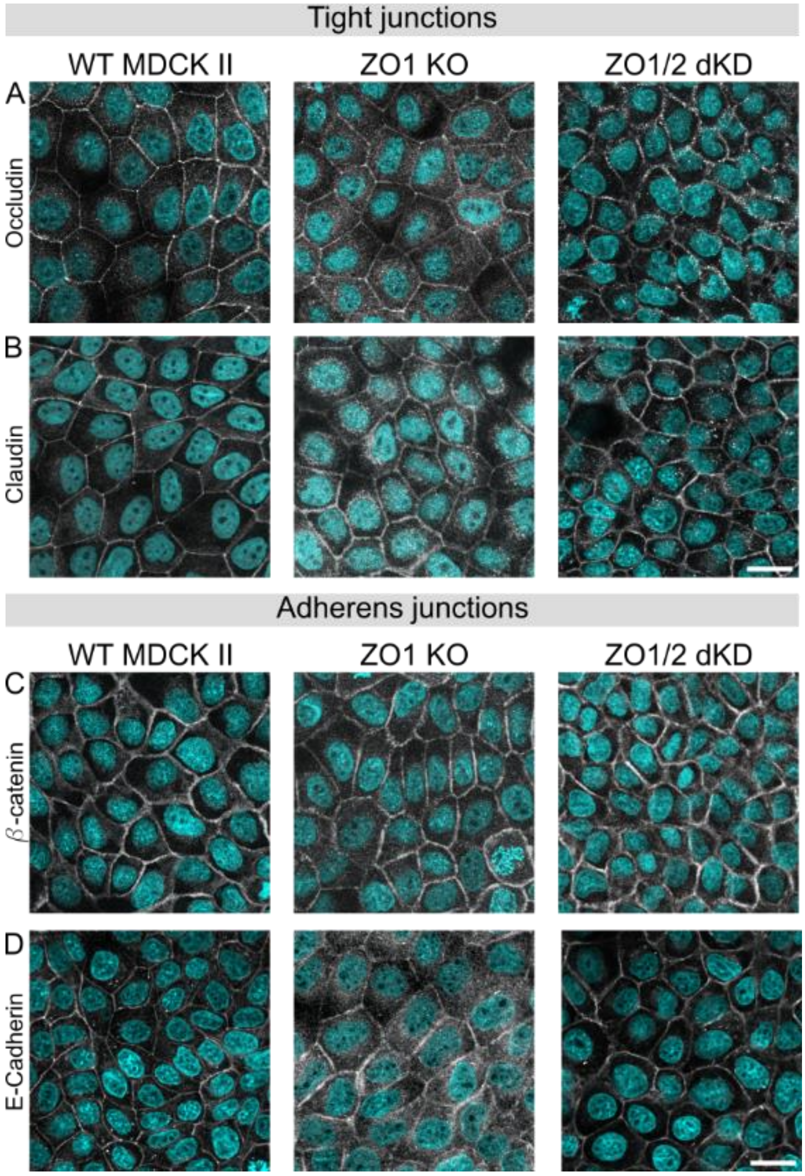

Figure S6. Immunofluorescence of tight junction transmembrane proteins and adherens junction proteins. A) Occludin. B) Claudin 1. C) β-catenin. D) E-Cadherin. Nuclei are shown in cyan. Scale bars: 20 µm.

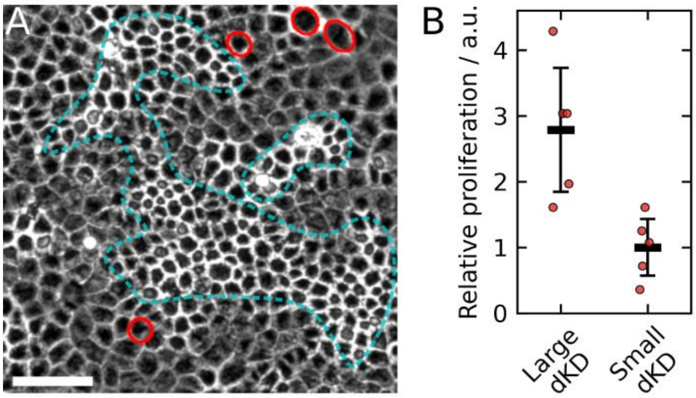

Figure S7. In the dKD monolayers, more of the large and stretched cells were observed to proliferate than the small and contractile cells. A) Example of dKD cells during migration with proliferation events indicated by red circles and patches of small cells highlighted in cyan. Scale bar: 50 µm. B) Relative proliferation of five example regions from A, normalized by the average number of small cell proliferation events. Proliferation events were counted and attributed by hand and the examples were chosen, so that approximately the same amount of large and small cells was present. These data were collected in the same time window as the MSD analysis, i.e., between 19 h and 21 h.
